# Menarche in primary ovarian insufficiency after a month of hormone replacement therapy: a case report

**DOI:** 10.1186/s13256-020-02603-6

**Published:** 2021-02-22

**Authors:** Biwen Cheng

**Affiliations:** Endocrinology Division, Department of Pediatrics, Hsinchu Mackay Memorial Hospital, No. 690, Section 2, Guangfu Rd., East District, Hsinchu, 300 Taiwan

**Keywords:** Primary ovarian insufficiency, Hormone replacement therapy, Menarche, Amenorrhea, Delayed puberty

## Abstract

**Background:**

Gynecologic anomalies, including uterine agenesis and ovarian dysgenesis, are some of the several differential diagnoses in adolescent females with primary amenorrhea and delayed puberty. Primary ovarian insufficiency is reported in the clinical practice of reproductive endocrinology can be determined by conducting sex hormone tests to evaluate the hypothalamic-pituitary-ovarian axis. However, confirmation of Mullerian agenesis by image modalities can be extremely challenging. Once the diagnosis is established, breakthrough bleeding usually occurs 2 to 3 years after hormonal replacement therapy.

**Case presentation:**

We report a case of a seventeen year old Taiwanese female, 46 XX karyotype, with ovarian dysgenesis and an initial tentative diagnosis of uterine agenesis who experienced a breakthrough bleeding after a month of hormonal replacement therapy.

**Conclusions:**

The breakthrough bleeding after a month of estrogen therapy in primary ovarian insufficiency is uncommon, and the diagnosis of the absent uterus can have an extensive psychological impact on patients and their families.

## Introduction

Primary ovarian insufficiency (POI) has previously been referred to as primary ovarian failure or premature menopause. The term POI was advocated by the National Institutes of Health, because the ovarian function in these individuals can be intermittent or unpredictable. It is, by definition, a loss of ovarian activity before the age of 40 years and characterized by irregular or absent periods and reduced fertility [1].

In young girls, the diagnosis of POI can be challenging. They present as delayed puberty and amenorrhea. The uterus or ovaries under image modalities may remain hidden, which in turn, complicates the diagnosis. Once POI is confirmed, hormonal replacement therapy is required for the induction of puberty to achieve secondary sexual characteristics and to reach an optimal final height with healthy bone mass. Thus, it can alleviate the initial psychological stress and may provide an opportunity for future fertility. A breakthrough bleeding can be expected 2 to 3 years after hormonal replacement therapy [2–4].

Here, we report a case of a child with ovarian dysfunction that experienced breakthrough bleeding after a month of estrogen therapy.

## Case presentation

A 17-year-old Taiwanese female presented to the gynecology clinic with complaints of primary amenorrhea and absence of secondary sexual characteristics. Her body height was 158.8 cm (40th percentile), weight was 45 kg (10th percentile), body mass index was 17.8 (normal range: 17.7–22.7 [5]) and normal intelligence. The patient’s mother and father had a body height of 149 cm and 175 cm with a mid-parental height estimate of 156 cm +/− 7 cm. She was born of consanguineous marriage with normal developmental milestones. She does not have any siblings.

**Fig. 1:**
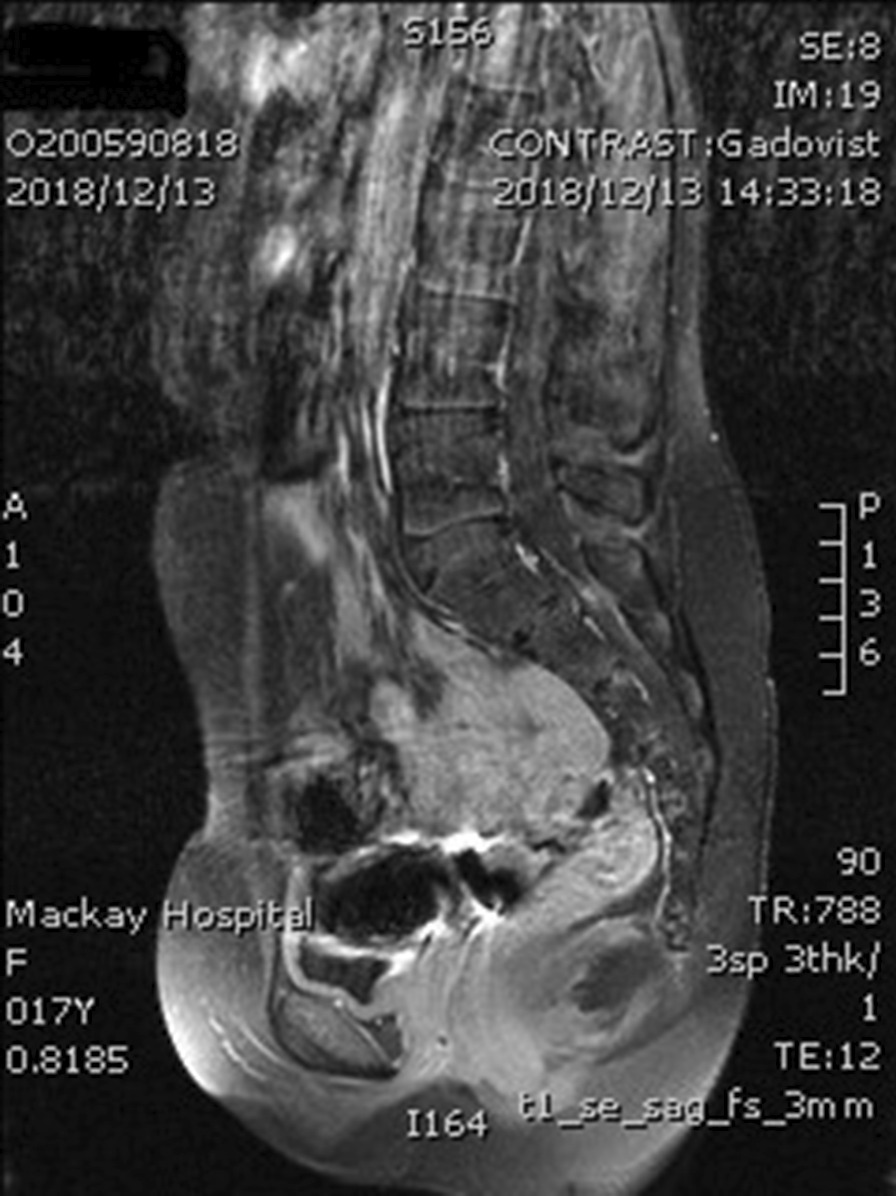
Absent of the uterus in magnetic resonance imaging

On physical examination, her cardiovascular, respiratory, and central nervous system showed no significant findings. She had pubic and axillary hair but had no breast bud development (Tanner stage B1PH3). On examination of the external genitalia, it was under-developed. Her estimated bone age was at 13 years, according to the Greulich and Pyle method. She received pelvic ultrasonography and magnetic resonance imaging (MRI), which both revealed the absence of the uterus and vaguely visible bilateral ovaries (Fig. 1). Laboratory investigation reported high random follicular-stimulating hormone/luteinizing hormone (FSH 58.89 mIU/mL, LH 20.46 mIU/mL) and estrogen (13 pg/mL) levels which indicated poor ovarian function. Prolactin levels and both renal and thyroid functions were normal. The patient referred to the pediatric endocrinology clinic for a suspected underlying endocrine disease.

At the pediatric endocrinology clinic, a Gonadotropin-receptor hormone stimulation test to verify ovarian function reported highly elevated FSH/LH (FSH peak 86.99 mIU/mL, LH peak 72.77 mIU/mL) levels and low estrogen level (<10 pg/mL). Karyotype report a normal female 46, XX genotype. The initial impression was ovarian agenesis and primary ovarian insufficiency (POI). A bone marrow density with dual-energy X-ray absorptiometry (DXA) skeletal survey assessment for osteoporosis reported a *Z*-score of − 2.7. The patient and family received comprehensive fertility counsel. The patient received hormone replacement therapy (HRT) with oral conjugated estrogen (0.625 mg/tablet) for the development of secondary sexual characteristics and restoring bone health.

**Fig. 2:**
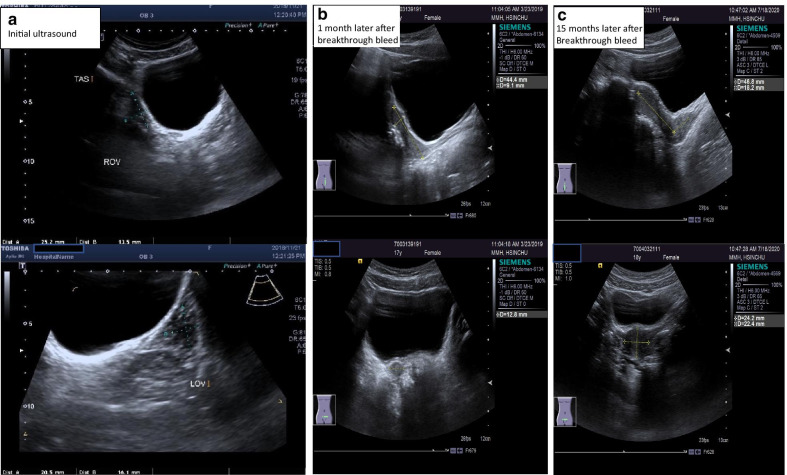
Uterine growth from undetectable (**A**) to detectable (**B**) to mature size (**C**) under estrogen replacement therapy. *ROV* right ovary, *LOV* left ovary

However, after a month of therapy, a breakthrough bleeding occurred. Follow-up pelvic ultrasonography reported a visible uterus [with a dimension of anterior-posterior (AP) × Length (*L*) × Transverse (*T*): 44 mm × 12 mm × 9 mm; Fig. 2] and physical examination also confirmed breast bud development. Primary ovarian insufficiency is, therefore, diagnosed. Therefore, oral estrogen (conjugated estrogen 0.625 mg) resumed with progesterone therapy (medroxyprogesterone 5 mg) applied. The family was relieved after receiving the news of the existing uterus. After one year of hormonal replacement therapy, both bone marrow density (BMD) (*Z* score from initial − 2.7 to − 1.8 ) and bone age maturation from the initial 13 to 15 years showed improvement. Her latest sonography reported a mature uterus (AP × *L* × *T*: 64 mm × 22 mm × 24 mm; Fig. 2.) with the continued development of secondary sex characteristics (Tanner stage B3Ph3) and regular menstruation.

## Discussion

Primary ovarian insufficiency is one of the leading causes of female infertility owing to an abnormal ovarian reserve. However, if it occurs at a younger age, the effect on growth and puberty can be devastating. Whether it is caused by follicle depletion or dysfunction, in 90% of cases, the etiology remains unknown [6, 7]. Near the age of eight-years uterine exposure to elevated estrogens stimulates growth, allowing the uterine size to progressively increases, especially at Tanner stages 3 and 4 [8]. If the ovarian function is absent, the uterus may be undetectable on imaging studies. Several publications have emphasized reaching the diagnosis of uterine agenesis must be made with absolute caution. In particular, the assessments are undertaken by non-experienced practitioners in adolescent gynecology [8–11]. In our patient, the MRI performed by our expert senior staff radiologist and the ultrasound by our gynecologist, we were not able to identify the uterus in the first place. For the MRI, the setting of sequential images under the thickness of 3 mm is, by standard, to be in detail. Assuming a thickness of 1 mm adjusted earlier, we may have detected the premature uterus initially. However, our radiologist advised that a 1 mm thickness per image sequences, its output produces an image quality that would have been a challenge for interpretation.

Our initial affirmation on the absence of the uterus made a strong emotional impact on our patient and her family. There are already publications warned of these intolerable impacts, in particular from the infertility aspect and perceived lack of psychosocial support in these individuals [12–15]. These patients often have a higher risk of major depression [12] with lower self-esteem [16] and higher social isolation [13]. Therefore we suggest that no conclusion should be made regarding the status of the uterus until adequate exposure to exogenous estrogen with repeat imaging are complete.

There is significant uncertainty about the appropriate timing and dosage for estrogen replacement therapy in the treatment of ovarian failure or dysfunction, especially in patients with Turner syndrome [17–19]. Both primary ovarian insufficiency and Turner syndrome have many similarities. For example, both have hypoestrogenism, which is a contributing factor to the low bone marrow density (BMD), and a significantly low bone mass for age on initial presentation [20, 21]. Estrogen replacement therapy (0.625 mg/day) is initiated to improve bone mass and to induce secondary sex characteristics [2]. Although the use of estrogen regimen and its timing is still an ongoing subject of debate, the current consensus is that if gonadotropin levels begin to elevate, pubertal induction with preferred low dose transdermal estrogen (1/4 to 1/2 patch) or oral conjugated/unconjugated estrogen (0.3 mg to 0.625 mg) should be initiated at the age of 11 to 12 [3, 22, 23]. The regimen is progressively titrated up to the full adult dosage to mimic normal physical and social development [3, 22, 23]. Breakthrough bleeding was reported to occur two to three years after a sustained period of estrogen replacement therapy in past studies [2–4]. However, pubertal induction commence by estrogen therapy in our patient was at a later age with a breakthrough bleeding occurred only after a month of estrogen treatment. We found no record of such occurrences in previous publications.

We hypothesize a surge of estrogen may have facilitated ovarian function, which then leads to the precipitous development of the uterus. A study in 2012 by Hewitt *et al.* demonstrated by injecting estrogen in rodent uterus, the binding of estrogen receptors alpha (ERα) to chromatin sites increases at least two folds, which facilitates uterine growth [24, 25]. In a few studies, more than half of young women with premature ovarian dysfunction can experience years of intermittent and unpredictable ovarian function [7, 26–30]. Administration of exogenous estrogen in combination with high levels of endogenous follicle-stimulating hormone (FSH) triggers the ovaries function that may have already been intermittent, which results in the fast growth of uterine tissue and endometrial lining. A very similar environmental situation in in-vitro fertilization (IVF). In IVF before follicle extraction, the gonadotropins are downregulated to achieve low levels of estrogen. It is then followed by adding FSH, which results in a concomitant estrogen surge and facilitates mature follicle development [31]. Further studies are required to confirm this suspicion. Of note, the haste uterine growth in our patient after a year of HRT reached a uterine length of 64 mm (AP), which is close to 65 mm commonly used to determine uterine maturity [32–35]. It is interesting that a recent study in 2019 by E.Burt *et al*. [36] reported uterine growth compromised in hypogonadism treated with HRT. This finding contrasts with our patient and past publications, where estrogen therapy can attain an adequate uterine size [37, 38]. Our patient contradicts the result of Burt *et al.* We report the first case of POI to experience menarche after a month of HRT with a fast-uterine growth.

## Conclusions

In summary, image modalities such as ultrasound and MRI may not be able to detect the presence of the uterus in prepubertal patients. Therefore, the diagnosis should be tentative and repeat imaging advised after a period of estrogen replacement. Currently, there is no clear explanation for the occurrences of breakthrough bleeding one month after HRT. The association between POI and abrupt menarche is unprecedented. Further studies regarding puberty induction in these individuals, as well as early breakthrough bleeding, are warranted.

## Data Availability

Data sharing not applicable to this article as no datasets were generated or analyzed during the current study.
